# Early [18]FDG PET/CT scan predicts tumor response in head and neck squamous cell cancer patients treated with erlotinib adjusted per smoking status

**DOI:** 10.3389/fonc.2022.939118

**Published:** 2022-08-30

**Authors:** Mercedes Porosnicu, Anderson O’Brien Cox, Joshua D. Waltonen, Paul M. Bunch, Ralph D’Agostino, Thomas W. Lycan, Richard Taylor, Dan W. Williams, Xiaofei Chen, Kirtikar Shukla, Brian E. Kouri, Tiffany Walker, Gregory Kucera, Hafiz S. Patwa, Christopher A. Sullivan, J. Dale Browne, Cristina M. Furdui

**Affiliations:** ^1^ Department of Internal Medicine, Section on Hematology and Oncology, Wake Forest School of Medicine, Atrium Health Wake Forest Baptist Medical Center, Winston-Salem, NC, United States; ^2^ Atrium Health Wake Forest Baptist Comprehensive Cancer Center, Winston-Salem, NC, United States; ^3^ Department of Otolaryngology, Wake Forest School of Medicine, Atrium Health Wake Forest Baptist Medical Center, Winston-Salem, NC, United States; ^4^ Department of Radiology, Wake Forest School of Medicine, Atrium Health Wake Forest Baptist Medical Center, Winston-Salem, NC, United States; ^5^ Department of Biostatistics and Data Science, Wake Forest School of Medicine, Atrium Health Wake Forest Baptist Medical Center, Winston-Salem, NC, United States; ^6^ Department of Internal Medicine, Section on Molecular Medicine, Wake Forest School of Medicine, Atrium Health Wake Forest Baptist Medical Center, Winston-Salem, NC, United States; ^7^ Department of Cancer Biology, Wake Forest School of Medicine, Atrium Health Wake Forest Baptist Medical Center, Winston-Salem, NC, United States

**Keywords:** head and neck cancer, erlotinib, [18FDG] PET/CT, smokers, women in science

## Abstract

**Translational Relevance:**

Evaluation of targeted therapies is urgently needed for the majority of patients with metastatic/recurrent head and neck squamous cell carcinoma (HNSCC) who progress after immunochemotherapy. Erlotinib, a targeted inhibitor of epidermal growth factor receptor pathway, lacks FDA approval in HNSCC due to inadequate tumor response. This study identifies two potential avenues to improve tumor response to erlotinib among patients with HNSCC. For the first time, this study shows that an increased erlotinib dose of 300 mg in smokers is well-tolerated and produces similar plasma drug concentration as the regular dose of 150 mg in non-smokers, with increased study-specific defined tumor response. The study also highlights the opportunity for improved patient selection for erlotinib treatment by demonstrating that early in-treatment [18]FDG PET/CT is a potential predictor of tumor response, with robust statistical correlations between metabolic changes on early in-treatment PET (4-7 days through treatment) and anatomic response measured by end-of-treatment CT.

**Purpose:**

Patients with advanced HNSCC failing immunochemotherapy have no standard treatment options. Accelerating the investigation of targeted drug therapies is imperative. Treatment with erlotinib produced low response rates in HNSCC. This study investigates the possibility of improved treatment response through patient smoking status-based erlotinib dose optimization, and through early in-treatment [18]FDG PET evaluation to differentiate responders from non-responders.

**Experimental design:**

In this window-of-opportunity study, patients with operable HNSCC received neoadjuvant erlotinib with dose determined by smoking status: 150 mg (E150) for non-smokers and 300 mg (E300) for active smokers. Plasma erlotinib levels were measured using mass spectrometry. Patients underwent PET/CT before treatment, between days 4-7 of treatment, and before surgery (post-treatment). Response was measured by diagnostic CT and was defined as decrease in maximum tumor diameter by ≥ 20% (responders), 10-19% (minimum-responders), and < 10% (non-responders).

**Results:**

Nineteen patients completed treatment, ten of whom were smokers. There were eleven responders, five minimum-responders, and three non-responders. Tumor response and plasma erlotinib levels were similar between the E150 and E300 patient groups. The percentage change on early PET/CT and post-treatment PET/CT compared to pre-treatment PET/CT were significantly correlated with the radiologic response on post-treatment CTs: R=0.63, p=0.0041 and R=0.71, p=0.00094, respectively.

**Conclusion:**

This pilot study suggests that early in-treatment PET/CT can predict response to erlotinib, and treatment with erlotinib dose adjusted according to smoking status is well-tolerated and may improve treatment response in HNSCC. These findings could help optimize erlotinib treatment in HNSCC and should be further investigated.

**Clinical Trial Registration:**

https://clinicaltrials.gov/ct2/show/NCT00601913, identifier NCT00601913.

## Introduction

Despite the recent advancements in the treatment of metastatic/recurrent head and neck squamous cell carcinoma (HNSCC), the patient prognosis remains inadequate. The current management of these patients is centered on the treatment with immune checkpoint inhibitors (ICI), while the investigation and application of targeted therapies lag behind. The response to the first line treatment is limited to 20% or less of patients when treated with single agent ICI, and 36% of patients when treated with concurrent ICI and chemotherapy (1). Targeted drug therapies are urgently needed for the subsequent-line treatment of patients with tumors that have progressed on both immunotherapy and chemotherapy.

Cetuximab, a monoclonal antibody that targets epidermal growth factor receptor (EGFR), is the only targeted therapy that the Food and Drug Administration currently approves to treat HNSCC as a radiation sensitizer for locoregionally advanced HNSCC (2, 3) or in combination with chemotherapy in metastatic/recurrent disease (4). Erlotinib, an orally administered EGFR inhibitor, is currently approved for the treatment of non-small cell lung cancer (NSCLC) and pancreatic cancer (5), but application in HNSCC was not pursued due to the poor efficacy reported by single-arm clinical trials. A phase II study of erlotinib (150 mg/day) in 115 patients with recurrent or metastatic HNSCC had a partial response (PR) rate of 4.3%, stable disease (SD) rate of 38.3%, median progression-free survival (PFS) of 2.2 months, and median overall survival (OS) of 6 months (6). A retrospective meta-analysis of EGFR tyrosine kinase inhibitors (TKI) (erlotinib, gefitinib and lapatinib) in 319 HNSCC patients enrolled in five studies, which included the OSI phase II study of erlotinib, found that the development of skin rash, better performance status, and older age were the only three factors associated with improved treatment benefit (7).

Active smoking has been investigated as a possible factor that may decrease tumor response to EGFR TKIs. A retrospective analysis from the National Cancer Institute of Canada Clinical Trials Group study BR.21a found a possible association between never-smoking status and treatment response to erlotinib 150 mg PO daily although it did not meet statistical significance (p = 0.054) (8). Similarly, a meta-analysis of NSCLC patients from nine and ten clinical trials with erlotinib and gefitinib, respectively, concluded that never smokers appear to show longer OS and PFS as compared to former or current smokers (9). At least part of this effect is due to the high prevalence of EGFR sensitizing mutations among never smokers, a confounding variable that this meta-analysis did not control, but it has been hypothesized that active smoking may also influence the metabolism of erlotinib. A retrospective analysis of serum samples from patients with solid tumors treated with erlotinib 150 mg daily showed that drug clearance in current smokers was 24% faster than the clearance in former smokers or never smokers (10). A study that administered erlotinib to healthy volunteers found that the geometric mean of the area under the curve (0-infinity) for the 150 mg dose was 2.8-fold lower among smokers than in non-smokers and similar to non-smokers who received the 300 mg dose (11). A follow-up phase III study of current smokers with NSCLC confirmed that higher dosing of erlotinib (300 mg daily, elected based on prior pharmacokinetic data (11)) achieved higher plasma concentrations than standard dosing (150 mg daily) but did not yield a significant difference in clinical outcomes (12). In addition to increased drug clearance, there may also be a direct effect of nicotine contributing to erlotinib resistance (13).

Another strategy to optimize treatment efficacy is by identifying early predictive factors for treatment response for a better patient selection. An early metabolic response on imaging with deoxy-2-[18F] fluoro-D-glucose ([18]FDG) positron emission tomography and computed tomography (PET/CT) may be able to predict treatment response to erlotinib. In a NSCLC study, an early metabolic response on PET imaging performed 48 hours after starting treatment with single agent erlotinib was found to significantly predict OS (14, 15). An *in vitro* study of HSNCC cell lines found that erlotinib inhibited extracellular signal-regulated kinase-1/2 (ERK-1/2) phosphorylation and was associated with reduction in [18]FDG uptake in animal and human tumors (16). Similarly, our *in vitro* investigations found increased sensitivity to erlotinib in HNSCC cells with higher glucose uptake (17, 18). Cumulatively, these reports suggest that PET imaging could serve as a potential early marker for response to erlotinib in HNSCC.

Window of opportunity studies may expedite drug development especially for targeted drug therapies, by administering short neoadjuvant treatments between diagnosis and surgical resection (19, 20). One such study administered erlotinib 150 mg daily for a median of 20 days in patients with HNSCC and found a 16% PR rate with no delays in surgery (21). Plasma concentrations of erlotinib and its metabolite OSI-420 demonstrated that erlotinib clearance was higher among smokers (median: 7.28 L/h) than non-smokers (4.98 L/h; p = 0.008) (22).

Thus, whereas smoking is a leading causative factor of HNSCC, it also appears to negatively affect efficacy of the TKI drugs inhibiting the EGFR pathway, predominantly targeted in the management of these patients. We hypothesized that the limited benefit previously reported with erlotinib treatment in patients with HNSCC could be improved by dosing the erlotinib according to smoking status. Therefore, we conducted a pilot window of opportunity study in patients with HNSCC scheduled for surgery and treated with erlotinib dosed at regular 150 mg (E150) for non-smokers or former smokers and at 300 mg (E300) for current smokers, and correlated tumor response with plasma erlotinib levels. Additionally, the study was designed to investigate early in-treatment PET as a possible predictor of erlotinib treatment response.

## Patients and methods

### Study design and eligibility criteria

This pilot study was conducted at the Wake Forest Baptist Medical Comprehensive Cancer Center (WFBMCCC; now Atrium Health WFBMCCC) was approved by the Institutional Review Board, and was registered with clinicaltrials.gov (NCT00601913). The study aimed to investigate the clinical and tissue effect and tolerance of a short course of erlotinib administered in a dose adjusted per smoking status as first line treatment before surgery and to investigate PET/CT scan as a possible early predictor of response to erlotinib. Patients were eligible if they had newly diagnosed, histologically confirmed HNSCC, defined as primary squamous cell carcinoma of the oral cavity, oropharynx, larynx, or hypopharynx, and were scheduled for surgical treatment. At least a 15-day window between the time of biopsy-proven diagnosis and surgery date was required to allow a minimum 14-days of planned treatment with erlotinib. The study excluded patients who have been treated before with systemic anti-cancer treatments or radiotherapy to the head and neck area. Biopsy samples at diagnosis and pathology samples from the surgical specimen, as well as weekly blood samples, were collected, processed, and stored at -80°C in a biospecimen repository.

### Treatment plan

Erlotinib dose was adjusted according to smoking status. Non-smokers and former smokers were treated with 150 mg erlotinib daily, while active smokers were treated with 300 mg erlotinib daily. Patients were defined as active smokers if they smoked more than ten cigarettes per day for more than one year and as former smokers if they were active smokers and quit more than one year before diagnosis. Patients were scheduled to take erlotinib daily for at least 14 days until the day before surgery. Erlotinib dosing was reduced or held for toxicity per protocol. Patients were followed for a period of 30 days after surgery for evaluation of treatment toxicities. Treatment toxicities were described and documented according to NCI Common Terminology Criteria for Adverse Events, version 3.0.

### Imaging evaluations

Patients underwent [18]FDG PET/CT evaluation fewer than 28 days before starting treatment and a diagnostic neck CT with contrast for determination of the largest tumor diameter fewer than 14 days before treatment. An early in-treatment PET/CT was performed between days 4-7 of erlotinib therapy, and then a post-treatment PET/CT for metabolic response and a diagnostic neck CT with contrast for tumor response measurement were performed the day before surgery. For descriptive analysis, patients were categorized as responders, minimum responders, and non-responders based on the change in maximum tumor diameter on the diagnostic neck CT. In the setting of window study design, with short duration of treatment, responders were defined as having a decrease in maximum tumor diameter ≥20%, minimum responders as having a decrease in maximum tumor diameter of 10-19%, and non-responders as having the maximum tumor diameter decreased by <10%, not changed, or increased, when the post-treatment diagnostic neck CT was compared to the pre-treatment diagnostic neck CT. For categorical statistical analysis, minimum responders were grouped with the non-responders. Metabolic response, expressed as percentage change in [18]FDG standardized uptake values (SUVs), was determined by comparing the early in-treatment PET/CT to the pre-treatment PET/CT to determine early metabolic response, and the post-treatment PET/CT to the pre-treatment PET/CT to determine post-treatment metabolic response. Patients with a decrease of >25% in PET/CT tumor SUV comparative with the pre-treatment PET/CT tumor SUV were considered responders (23).

### Quantitative measurements of plasma erlotinib

#### Materials

Water, methanol (MeOH), acetonitrile, formic acid, and potassium hydroxide were purchased from ThermoFisher Scientific (Waltham, Massachusetts). Erlotinib was purchased from Cayman Chemical (Ann Arbor, Michigan) and OSI-420 was purchased from Selleckchem (Houston, Texas).

#### Extraction and quantitative analysis by targeted liquid chromatography mass spectrometry (LC-MS/MS)

A volume of 100 μL of plasma was added to 400 μL of ice-cold methanol, vortexed and incubated for one hour on ice. The extract was then centrifuged at 18,000 x g for 5 minutes at 4°C. The supernatant was collected and dried under vacuum. The resulting residue was reconstituted in 100 μL MeOH, sonicated, and centrifuged a second time with the same parameters. The supernatant was collected and analyzed by LC-MS/MS after 100-fold dilution in water with 0.1% formic acid. Gradient separation and analysis of erlotinib and its metabolite OSI-420 was performed using a Shimadzu Nexera UHPLC system in tandem with a Shimadzu 8050 triple-quadrupole mass spectrometer utilizing a DUIS source. The mobile phase system was made up of water with 0.1% formic acid (mobile phase A) and acetonitrile with 0.1% formic acid (mobile phase B). An Agilent Zorbax Eclipse Plus C18 RRHD column was employed for the separation. The solvent gradient system consisted of a 4 min isocratic flow at 35% B followed by a ramp to 95% B ending at 5 minutes, a hold at 95% B until 8 minutes, and finally another isocratic flow at 35% B from 8.1 minutes to 12 minutes. Erlotinib and OSI-420 were measured in positive mode using the following MRM transitions: Erlotinib 393.50 > 278.10, 336.15; OSI-420 380.00 > 278.10, 322.1 0 using established procedures (24). The quantification was performed using external standard calibration with erlotinib and OSI-420.

### Statistical analyses

Descriptive analyses were performed by erlotinib dose for patient demographic variables, tumor characteristics, treatment length, tobacco and alcohol use, and toxicities. We examined the correlation between the percentage of early metabolic (i.e., PET/CT) response or post-treatment metabolic response and the percentage of anatomic (i.e., neck CT) tumor response, as well as the correlations between the erlotinib plasma content as measured by LC-MS/MS and percentage of anatomic tumor response and other analyses, using Pearson Correlations in R (25). Graphics were generated with ggplot2 (26). Next, we created binary variables for responders/non-responders using an anatomic cut-point of 20% for neck CT (minimum responders were grouped with non-responders) and a metabolic cut-point of 25% for PET/CT tumor SUV change. With these cut-points, we created two 2 by 2 tables to examine the sensitivity and specificity of the PET/CT for predicting anatomic response as measured by post-treatment neck CT.

## Results

Twenty-four patients were enrolled in the study, 23 patients with HNSCC of the oral cavity and one patient with HNSCC of the larynx. Patients were numbered according to **Table 3**. One patient (patient 24) was removed from the study before starting treatment due to non-compliance and was excluded from all analyses. The demographic data and disease characteristics for the remaining 23 patients are presented in **Table 1**.

**Table 1:** Patient demographics by erlotinib dose.

		All participants		Dose = 150		Dose = 300	
		n	% or mean	n	% or mean	n	% or mean
Age		23	59.9	11	61.6	12	58.3
Gender	*Male*	15	65.2	4	36.4	11	91.7
	*Female*	8	34.8	7	63.6	1	8.3
Tumor at diagnosis	*T2*	6	26.1	4	36.4	2	16.7
	*T3*	4	17.4	2	18.2	2	16.7
	*T4*	13	56.5	5	45.5	8	66.7
Node at diagnosis	*N0*	10	43.5	6	54.5	4	33.3
	*N1*	3	13.0	0	0.0	3	25.0
	*N2b*	5	21.7	2	18.2	3	25.0
	*N2c*	5	21.7	3	27.3	2	16.7
Stage	*II*	4	17.4	3	27.3	1	8.3
	*III*	2	8.7	1	9.1	1	8.3
	*IV*	17	73.9	7	63.6	10	83.3
Tobacco use	*Current*	14	60.9	2^a^	18.2	12	100.0
	*Former*	6	26.1	6	54.5	0	0.0
	*Never*	3	13.0	3	27.3	0	0.0
Alcohol use	*Yes*	12	52.2	2	18.2	10	83.3
	*No*	11	47.8	9	81.8	2	16.7
Treatment days^b^		19	18.3	9	17.7	10	19.0

a Tobacco chewers – unable to quantify.

b For the 19 patients analyzed for treatment efficacy.

Of the 23 patients, four did not complete treatment and evaluations as planned per clinical protocol (patients 20-23). They were maintained in the toxicity analysis but excluded from the treatment response analysis. Two of the four patients (patients 20 and 21) declined to finish treatment and to have final imaging evaluation after completing 12 and 15 days, respectively, of the planned 17 days of treatment with erlotinib, and they proceeded with surgery as scheduled. The other two of the four patients (patients 22 and 23) were hospitalized with cancer related complications (aspiration pneumonia). They did not proceed with surgery as initially scheduled and were removed from the protocol after 11 and 4 days respectively of treatment with erlotinib. Three of these four patients who were treated for longer than 6 days had the early in-treatment PET/CT performed.

Nineteen patients completed protocol treatment and were analyzed for response. One patient (patient 6) declined to have surgery but had all imaging evaluations performed as scheduled, and collection of the surgical specimen was replaced with a biopsy at the end of treatment. All 19 patients underwent the early in-treatment PET/CT evaluation. The 19 patients were treated with erlotinib for an average of 18.3 (range 14-27) days. Ten patients were classified as smokers and were treated per protocol with E300 for an average of 19 (range: 14-27) days. Nine patients were considered non-smokers (including former smokers and never smokers) and were treated with E150 mg for an average of 17.7 (range: 14-26) days. Three of these nine patients chewed tobacco in an unquantified amount.

### Toxicity analysis

All 23 enrolled patients had data analyzed for toxicity (**Table 2**). There were no delays in the planned surgery and no erlotinib-related complications post-surgery. Four patients had treatment alterations due to toxicity. Two patients (patients 20 and 21) made a personal decision to stop treatment after 12 and 15 of the planned days of treatment due to skin rash grade I and grade II, respectively. Patient 12 developed grade III skin rash 16 days after beginning treatment with E300. He received a reduced dose of 200 mg erlotinib for four days and then stopped treatment two days before surgery when he did not show improvement. Another patient (patient 7), current tobacco chewer, developed grade 3 mucositis after 11 days of treatment with E150, and the treatment was discontinued for the remaining 3 days before surgery. This participant was also non-adherent with clinician recommendations and lost significant weight. There were no other grade III toxicities. For a full summary of patient toxicities, see **Table 2**.

**Table 2:** Patient toxicities by erlotinib dose.

		Dose = 150 (N = 11)		Dose = 300 (N = 12)	
Complication	Grade	n	%	n	%
Rash	*1*	3	27.3	6	50.0
	*2*	5	45.5	5	41.7
	*3*	0	0.0	1	8.3
Diarrhea	*1*	3	27.3	5	41.7
	*2*	1	9.1	0	0.0
Nausea	*1*	4	36.4	3	25.0
Mucositis	*2*	2	18.2	0	0.0
	*3*	1	9.1	0	0.0
Dry eyes	*1*	2	18.2	0	0.0
Loss of appetite	*1*	0	0.0	2	16.7
Elevated bilirubin	*1*	3	27.3	2	16.7
	*2*	3	27.3	2	16.7
Elevated GGT	*1*	2	18.2	1	8.3
Elevated Alkaline Phosphatase	*1*	0	0.0	1	8.3
Elevated SGOT	*1*	0	0.0	4	33.3
Elevated SGPT	*1*	0	0.0	2	16.7
	*2*	0	0.0	1	8.3
Decreased Mg	*1*	4	36.4	9	75

### Treatment response

Imaging and analytical results for all patients are included in **Table 3**. Nineteen patients had tumors measured by neck CT before and after the treatment. There were 11 responders, 5 minimum responders, and 3 non-responders.

**Table 3:** Summary of clinical and laboratory measurements.

Patient	Dose Erlotinib (mg)	Duration Treatment Planned	Duration Treatment Received	Neck CT% change in max. diameter	Day of Early In-Treatment PET/CT	Early In-Treatment PET/CT % change in SUV	Post-treatment PET/CT % change in SUV	Erlotinib (ng/mL)	OSI-420 (ng/mL)
1	150	17	17	-37	7	-26	-26	NIA	N/A
2	150	20	20	-28	6	-28	-10	3407.6	82.75
3	150	14	14	-22	4	-27	-43	7520.85	533.1
4	150	15	15	-21	6	-38	-55	NIA	N/A
5	150	21	21	-21	6	-52	-37	113.95	2.65
6	150	26	26	-16	6	-32	-40	1635.35	83.25
7	150	14	11	-13	6	-13	NIA	NIA	N/A
8	150	14	14	-10	6	-27	-12	163ll.15	22.5
9	150	18	18	0	6	5	+9	706.85	14.65
10	300	27	27	-45	6	-43	-70	6023.4	166.05
11	300	15	15	-27	5	-70	-65	3753.15	46.25
12	300	22	20	-25	6	-39	-49	325.8	0.5
13	300	18	18	-24	7	-43	-43	NIA	N/A
14	300	14	14	-21	6	-56	-52	3955.8	105.55
15	300	16	16	-21	6	-25	-32	1964.9	33.2
16	300	20	20	-10	6	-25	-25	3172.05	107.65
17	300	21	21	-10	6	-25	-20	598.85	0.75
18	300	25	25	0	6	+84	67	NIA	N/A
19	300	14	14	0	6	-2	-5	212.35	0

20	150	17	12	-20 (at 6 days)	6	-54	refused	1170.75	5.65
21	300	17	15	-13 (at 6 days)	6	-54	refused	3775.85	101.75
22	300	17	11	Not measurable	9	-52	off protocol-no longer a candidate for surgery	N/A	
23	150	15	4	Removed from the protocol due to tumor complications				N/A	
24	Removed from the protocol due to non-compliance							N/A	

Pink - patients receiving 150 mg erlotinib; Cyan - patients receiving 300 mg erlotinib; Gray- patients without post-treatment PET/CT. N/A Not Available.

**Figure 1:**
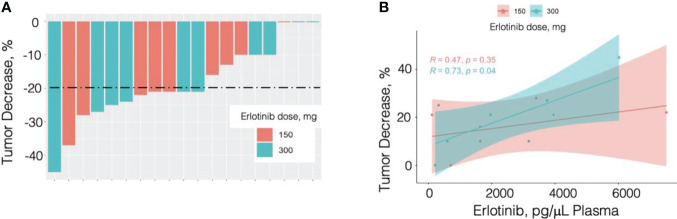
Percent tumor response by patient and dose **(A)** and by plasma erlotinib measured by LC/MS/MS analysis **(B)**. N = 19; 10 patients received erlotinib at 300 mg PO daily (cyan in panel **A** and panel **B**) and 9 received erlotinib 150 mg PO daily (pink in panel **A** and panel **B**).

Response profile was very similar between the E150 and E300 patient groups. Among the 10 patients in the E300 group there were 6 responders, 2 minimum responders, and 2 non-responders. Among the 9 patients in the E150 group there were 5 responders, 3 minimum responders, and 1 non-responder (**Figure 1A**).

One patient (patient 6) in the E150 group considered to be a minimum responder per diagnostic neck CT tumor measurements was a patient with Fanconi’s anemia. Her tumor was large and diffusely infiltrating, rendering neck CT tumor measurement challenging. Thus, only measurements from part of the tumor were used to define response. Clinically, this patient did well with a decreased need for pain medications and an improvement in performance status. She cancelled the scheduled palliative surgery for tumor debulking and underwent a biopsy instead and the end of treatment PET/CT. Given the clinical benefit, she continued treatment with erlotinib off protocol.

Two other patients were not analyzed for tumor response because they declined to undergo post-treatment imaging (patients 20 and 21). However, both patients underwent early in-treatment PET/CT, which showed a 54% decrease in tumor SUV in both patients and a 20% and a 13% decrease in tumor maximum diameter as measured on the attenuation correction CT component of the early in-treatment PET/CT.

### Correlation of anatomic tumor response with PET/CT metabolic results

**Figure 2:**
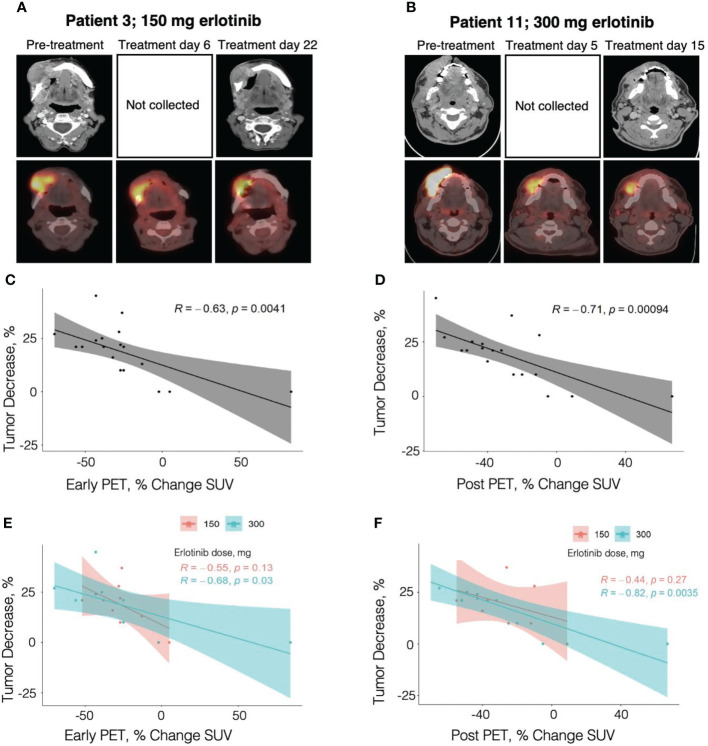
Correlation of percent change in tumor diameter with percent change in PET/CT SUV. **(A)** Contrast-enhanced neck CT and PET/CT images obtained in an 82 year-old female patient with OC T4N0M0, former smoker, treated with erlotinib dose of 150 mg. **(B)** Contrast-enhanced neck CT and PET/CT images obtained in a 55 year-old male patient with OC T2N2bM0, active smoker, treated with erlotinib dose of 300 mg. **(C)** - **(F)** Pearson correlation analysis of tumor diameter percent change with the percent change in SUV at early in-treatment PET/CT **(C** and **E,** 19 patients**)** and at post-treatment PET/CT **(D** and **F,** 18 patients**)**. Panels **(C)** and **(D)** present the analysis across the smokers and non-smokers combined, while panels **(E)** and **(F)** show correlation analysis for each group. .

PET/CTs were obtained before treatment, early during treatment (day 4-7), and after treatment with erlotinib in all 19 patients analyzed for treatment response, except one patient (patient 7) who refused the post-treatment PET/CT. The percentage change in the early in-treatment PET/CT and the post-treatment PET/CT scan tumor SUVs compared to the pre-treatment PET/CT tumor SUVs were found to be significantly correlated with the diagnostic neck CT anatomic tumor response in the 19 and 18 patients, respectively (R=0.63, p=0.0041; and R=0.71, p=0.00094, respectively) (**Figures 2A–F**).

#### Early in-treatment PET/CT

Twelve of the 19 patients (63%) had a decrease in the early in-treatment PET/CT relative to the pre-treatment PET/CT tumor SUV of >25%. Among responders, 10 out of 11 patients (90.9%) had concordance between the post-treatment anatomic tumor response as determined by diagnostic neck CT and the early in-treatment PET/CT metabolic response. Of the minimum responders and non-responders, 6 out of 8 patients (75%) had concordance with early in-treatment PET/CT metabolic results. Thus, the sensitivity and specificity of early in-treatment PET/CT for predicting post-treatment anatomic tumor response were 90.9% and 75%, respectively. One of the two discordant patients is the patient with Fanconi anemia (patient 6) who was deemed as a minimum responder based on the challenging neck CT measurements but who had a 32% early in-treatment PET/CT metabolic response, concordant with the good clinical response to erlotinib. The early in-treatment PET/CT SUV percent changes were as follows: -25 to -70% (mean 40.6%) in responders, -13 to -32% (mean 24.4%) in minimum responders, and -2% to +84% in non-responders. Overall, anatomic tumor response by neck CT correlated strongly with the SUV percent change on early in-treatment PET/CT (R=0.63, p=0.0041) with the strongest contribution from the E300 smoker group (R=0.68, p=0.03), while the correlation within the E150 non-smoker group was not significant (R=0.55, p=0.13) (**Figures 2C–E**).

#### Early in-treatment PET/CT - patients not included in the efficacy analysis

All three patients who completed the early in-treatment PET/CT but did not complete the post-treatment evaluations had an early in-treatment PET/CT metabolic response of -52% to -54% change in SUV. One patient (patient 20) in the E150 group who received treatment for just 6 days had a reduction in the maximum tumor diameter of 20% as measured on the attenuation correction CT component of the early in-treatment PET/CT and a metabolic response of -54% change in SUV from pre-treatment baseline. Another patient (patient 21) in the E300 group who also received treatment for 6 days, had a metabolic response of -54% on the early in-treatment PET/CT and a decrease in the maximum tumor diameter of 13% as measured on the attenuation correction CT of the same PET/CT. Surgical pathology measurements confirmed a significant decrease in size relative to the tumor measurements on the pre-treatment diagnostic CT. The third patient (patient 22) had the early in-treatment PET/CT delayed to day 9 due to sepsis. He displayed a -52% metabolic response, which corresponded with the significant clinical decrease size of the oral cavity mass, as well as decrease in pain.

#### Post-treatment PET/CT

Eleven of the 18 patients (61%) had a decrease in post-treatment PET/CT relative to the pre-treatment PET/CT tumor SUV of >25%. The percent metabolic response on the post-treatment PET/CT similarly displayed a high concordance with anatomic tumor response measured by diagnostic neck CT. Among responders, 10 out of 11 patients (90.9%) had concordance with post-treatment PET/CT metabolic response. The discordant patient (patient 2) had a concordant response on early in-treatment PET/CT, but only a 10% decrease in tumor SUV from baseline was observed on post-treatment PET/CT. Among minimum responders and non-responders, 6 out of 7 patients (85.7%) had concordance with the post-treatment PET/CT metabolic response. The discordant patient from this group was the patient with Fanconi Anemia (patient 6) who also had discordance on early in-treatment PET/CT. As previously discussed, the PET/CT metabolic responses were concordant with the observed clinical improvement in the context of difficult neck CT tumor measurements. The post-treatment PET/CT SUV percent changes were as follows: -10 to -70% (mean 48.2%) in responders, -12 to -40% (mean 24.2%) in minimum responders. and -5% to +67% in non-responders. There was a stronger correlation of the anatomic tumor response measured by neck CT with the post-treatment PET/CT metabolic response (R=0.71, p=0.00094) than with the early in-treatment PET/CT metabolic response (R=0.63, p=0.0041) (**Figures 2C, D**). Correlations remained statistically significant in the E300 group (post-treatment PET/CT R=0.82, p=0.0035; early in-treatment PET/CT R=0.68, p=0.03) but not in the E150 group (post-treatment PET/CT R=0.44, p=0.27; early in-treatment PET/CT R=0.55, p=0.13) (**Figures 2E, F**).

### Correlation of anatomic tumor response with erlotinib concentration in the blood

Erlotinib and its metabolite OSI-420 were measured in the plasma collected in the last day of treatment. using targeted LC-MS/MS analysis as described in *Materials and Methods*. Erlotinib concentration was on average 2500.8 ng/mL in the E300 group and 2503.8 ng/mL in the E150 group. As the OSI-420 concentration represented a small fraction of erlotinib concertation levels (<5%), the statistical analysis was performed using plasma erlotinib values. Plasma erlotinib showed a significant correlation with the response to treatment (**Table 3**; R=0.592, p=0.026). Breakdown of the analysis for the smoker and non-smoker groups highlighted, however, a stronger and statistically significant correlation in the E300 smoker group (R=0.73, p=0.04), while the correlation in the E150 non-smoker group was not statistically significant but followed the same trend (R=0.47, p=0.35) (**Figure 1B**).

## Discussion

EGFR is the only targeted signaling pathway with FDA approved treatment in patients with HNSCC. However, previous studies with single agent EGFR TKIs have reported consistent limited response rates of 5–15% in unselected HNSCC patients (7). Identification of predictors of response for improved patient selection and investigation of treatment dose and pharmacokinetics are established approaches to treatment optimization. Aligned with these goals, this pilot study investigated the value of early in-treatment PET/CT as a predictor of anatomic tumor response in patients with HNSCC undergoing erlotinib therapy. In addition, this is the first study to evaluate the tolerance and efficacy of erlotinib when dose adjusted according to smoking status in patients with HNSCC.

The window of opportunity study design allows for investigating the study hypothesis on a small cohort of previously untreated HNSCC patient population, eliminating potential interfering factors from prior anti-cancer treatments and thus allowing for an unbiased treatment setting for uncovering level and mechanisms of drug activity as well as predictors of response (21, 27–29).

The study presented here showed that it is feasible to treat HNSCC with erlotinib dose adjusted according to the smoking status. Twelve out of 23 patients were current smokers and treated with the erlotinib 300 mg daily dose (E300), with the other 11 patients treated with the 150 mg dose (E150). Independent of the erlotinib dose, the treatment was well tolerated. The incidence of significant toxicities was similar in the two groups. Four patients (two from each dose group) discontinued erlotinib treatment early. Two patients elected to stop treatment because of grade I and II skin rash. The other two patients (one from each dose group) discontinued treatment because of grade 3 toxicities (skin rash and mucositis). The only toxicities found more frequently in the E300 group were grade I skin rash, grade I increase in SGOT and SGPT, and grade I decrease in magnesium level (**Table 2**).

Erlotinib dose adjusted according to smoking status in first line treatment of patients with HNSCC demonstrated ability to induce an early response to treatment. Eleven of 19 (58%) treated patients had a decrease in maximum tumor diameter of more than 20% after an average of 20 days of treatment. Among responders, patients treated for more days experienced a greater decrease in tumor diameter. For example, the patients with decrease in tumor diameter of >25% had a 20 days average treatment duration, compared to 16 days for patients with decrease in tumor diameter of 20-25%. This finding suggests that an improved response could be achieved with continuation of treatment. The high percentage of responders found in this study relative to previously reported response rates may relate to this study-specific definition of response, in which a 20% decrease in the maximum tumor diameter of a single target lesion was selected as the lower limit of response instead of the standard RECIST 1.1 definition. Other window design protocols, in the same treatment-naïve HNSCC patient population, exhibited similar high responses. Day et al. (30) reported that 14 of 16 patients treated with Rapamycin displayed tumor shrinkage. In Schmitz et al. study (31), eight out of fourteen patients treated with cetuximab had a decrease in the largest tumor diameter of 8-30%. The effect of erlotinib as single agent or in combination with the Src inhibitor dasatinib was investigated by Gross et al. (28) and showed significantly increased response comparative with dasatinb single agent or placebo (p=0.0014).

Plasma levels of erlotinib and its metabolite OSI-420 quantified by LC-MS/MS were within the range reported by other studies (6, 12). While the erlotinib concentrations were found higher in the E300 treated patients in a previous phase III randomized NSCLC study (12), erlotinib concentrations measured in plasma by LC-MS/MS in this study were impressively similar between the E300 smoker group (average erlotinib concentration of 2500.8 ng/mL) and the E150 non-smoker group (average erlotinib concentration of 2503.8 ng/mL). This finding, in concordance with similar tumor response rates as measured by CT imaging, supports the concept of dosing erlotinib according to smoking status in the management of patients with HNSCC.

Five patients (5, 9, 12, 17, and 19) showed low plasma levels of erlotinib and OSI-420 (patients 5 and 9 in the E150 and the remaining three in the E300 group). To rule out analytical errors in the erlotinib level measurements and to account for potential PK/PD differences, we quantified OSI-420, a key metabolite of erlotinib. The proportion of OSI-420 was consistent across patients and aligned with other reports (6, 12), confirming the rigor of our methods. Study medication non-compliance is likely for three patients (9, 17, and 19), who showed complete lack of tumor response (9 and 19) or limited response (17) based on the neck CT imaging evaluation. Interestingly, two patients (5 and 12) demonstrated good anatomic responses to treatment despite low plasma erlotinib at the time of measurement. This finding can be explained either by study medication non-compliance at the end of treatment before final blood samples collection, or, alternatively, there is the possibility of increased tumor sensitivity to erlotinib among these patients. At the other end of the spectrum, two patients (3 and 10; in the E150 and E300 groups, respectively) had the highest plasma erlotinib levels (7520.85 and 6023.40 ng/mL, respectively), and they were the only patients showing significant increase in PET/CT metabolic response with further treatment (patient 3 from -27% to -43%, and patient 10 from -43% to -70%), possibly indicating more favorable pharmacokinetics.

Finally, this study suggests that an early in-treatment PET/CT obtained after 4-7 days of treatment can predict anatomic tumor response to erlotinib. Of 19 patients treated, 12 patients (63%) displayed a decrease in early in-treatment PET/CT SUV of > 25%. There was a significant correlation (R=0.63, p=0.0041) between metabolic response observed on early in-treatment PET/CT and anatomic tumor response measured by post-treatment diagnostic neck CT imaging. The early in-treatment PET/CT showed a sensitivity of 90.9% and a specificity of 75% in predicting tumor anatomic response. Importantly, the SUV percent change between the early in-treatment PET/CT and the post-treatment PET/CT was remarkably small (on average 5%), suggesting that, among erlotinib responders, the greatest metabolic response (as measured by SUV percent change) occurs in the first few days of erlotinib therapy with further decrease in metabolic activity occurring slowly over the remaining duration of therapy. Thus, early in-treatment PET/CT appears to be an excellent early predictor of post-treatment anatomic tumor response to erlotinib in HNSCC, consistent with findings in NSCLC patients (32). Other targeted drugs tested in in patients with HNSCC displayed remarkable rates of metabolic response defined as > 25% decrease in PET SUV, measured after short-duration treatment in similar window setting: 18 of 19 patients treated with cetuximab in Schmitz et al. study (31), 16 of 23 patients treated with the irreversible ErbB inhibitor afatinib in Machiels et at study (33) and six of 13 patients treated with the MEK inhibitor trametinib in Uppaluri et al. study (34). Although a direct correlation between the metabolic response measured by [18]FDG PET and the anatomic response measured by diagnostic CT or MRI scans was not attempted in the cited studies, these results, collectively, support the consideration for further evaluation of the [18]FDG PET as an early predictor, able to differentiate responders from non-responders in the treatment with some targeted biologic agents.

While this study challenged the limitations of treatment with erlotinib in HNSCC by exploring early in-treatment PET/CT as a predictor of response and by proposing a modified erlotinib treatment dosing based on the smoking status of the patient, the findings are limited by the short duration of treatment, the low number of patients, and the absence of control groups. It should also be noted that the presented erlotinib treatment results were evaluated in first line treatment of early stages of HNSCC. The findings presented by this pilot study have hypothesis-generating significance, recommending large confirmatory studies, both in early curative stages, as well as in advanced palliative treatment.

In conclusion, this pilot study is the first to show that HSNCC patient smoking status-based erlotinib dose adjustment is well tolerated and may increase the rate of tumor response. Although the daily dose of erlotinib was doubled in smokers, the blood concentration of erlotinib was similar to non-smokers. This finding supports the hypothesis that the standard dose of erlotinib, was sub-therapeutic for the majority of HNSCC patients who were active smokers. Furthermore, this study described the early in-treatment PET/CT as a possible predictor of tumor response to erlotinib in HNSCC. A strong early metabolic response was described on the in-treatment PET/CT, with significant statistical correlation with the anatomic response measured by the diagnostic neck CT at the end of treatment. A prospective large confirmatory study could utilize the results of early in-treatment PET/CT performed after the first week of treatment to determine which patients should continue erlotinib therapy and which patients should discontinue erlotinib and consider alternative therapeutic options. Further investigation and optimization of erlotinib and other targeted drug treatments are urgently needed for the majority of patients with metastatic/recurrent HNSCC who fail immunochemotherapy and for whom no standard treatment options are available.

## Data availability statement

The raw data supporting the conclusions of this article will be made available by the authors, without undue reservation.

## Ethics statement

The studies involving human participants were reviewed and approved by Institutional Review Board of Wake Forest University Baptist Medical Center, Winston Salem, North Carolina, United States. The patients/participants provided their written informed consent to participate in this study.

## Author contributions

Conception and methodology: MP, PB, RD. Data curation and investigation: MP, JW, PB, TL, RD, DW, BK, TW, GK, HP, CS, JB, AC, KS, XC, CF. Formal analysis: MP, JW, PB, RD, TL, DW, AC, KS, XC, CF. Supervision and project administration: MP, CF. Statistical analysis: MP, RD, CF. Writing the original draft: MP, RD, RT, AC, XC, CF. Visualization: MP, PB, RT, DW, AC, CF. All authors contributed to manuscript revision, read, and approved the submitted version.

## Funding

MP received funding support from Astellas Pharma for translational studies to be performed on tissue, blood and saliva collected from patients enrolled in the protocol. Astellas Pharma was not involved in the study design, collection, analysis, interpretation of data, the writing of this article or the decision to submit it for publication.

## Acknowledgments

The project described was supported by the National Center for Advancing Translational Sciences (NCATS), National Institutes of Health, through Grant Award Number UL1TR001420, and NIH/NCI U01CA215848 (PI Furdui/Kemp). This study was supported by the Wake Forest Baptist Compressive Cancer Center’s Tumor Tissue and Pathology Shared Resource and Proteomics and Metabolomics Shared Resource, funded by the National Cancer Institute’s Cancer Center Support Grant award number P30CA012197. The content is solely the responsibility of the authors and does not necessarily represent the official views of the NIH. In addition, the authors would like to acknowledge Mac Robinson, Ph.D. for help in editing the document.

## Conflict of interest

The authors declare that the research was conducted in the absence of any commercial or financial relationships that could be constructed as a potential conflict of interest.

## Publisher’s note

All claims expressed in this article are solely those of the authors and do not necessarily represent those of their affiliated organizations, or those of the publisher, the editors and the reviewers. Any product that may be evaluated in this article, or claim that may be made by its manufacturer, is not guaranteed or endorsed by the publisher.
